# Pretreatment Metabolic Parameters Measured by ^18^F-FDG PET to Predict the Pathological Treatment Response of HCC Patients Treated With PD-1 Inhibitors and Lenvatinib as a Conversion Therapy in BCLC Stage C

**DOI:** 10.3389/fonc.2022.884372

**Published:** 2022-06-03

**Authors:** Guanyun Wang, Wenwen Zhang, Jiaxin Chen, Xiaohui Luan, Zhanbo Wang, Yanmei Wang, Xiaodan Xu, Shulin Yao, Zhiwei Guan, Jiahe Tian, Shichun Lu, Baixuan Xu, Guangyu Ma

**Affiliations:** ^1^ Department of Nuclear Medicine, The First Medical Centre, Chinese the People's Liberation Army (PLA) General Hospital, Beijing, China; ^2^ Key Laboratory of Digital Hepetobiliary Surgery, Faculty of Hepato-Pancreato-Biliary Surgery, Chinese People's Liberation Army (PLA) General Hospital, Institute of Hepatobiliary Surgery of Chinese People's Liberation Army (PLA), Beijing, China; ^3^ Department of Oncology, The Fifth Medical Centre, Chinese People's Liberation Army (PLA) General Hospital, Beijing, China; ^4^ Graduate School, Medical School of Chinese People's Liberation Army (PLA), Beijing, China; ^5^ Department of Pathology, The First Medical Centre, Chinese People's Liberation Army (PLA) General Hospital, Beijing, China; ^6^ General Electric (GE) Healthcare China, Shanghai, China

**Keywords:** hepatocellular carcinoma, BCLC stage C, conversion therapy, pathological treatment response, positron emission tomography

## Abstract

**Objectives:**

This study aimed to assess the pretreatment ^18^F-fluorodeoxyglucose positron emission tomography/computed tomography (^18^F-FDG PET/CT) as a predictor of the pathological treatment response (PTR) of hepatocellular carcinoma (HCC) patients treated with PD-1 inhibitors and lenvatinib as a conversion therapy in BCLC stage C.

**Methods:**

All patients (n=20) underwent pretreatment ^18^F-FDG PET/CT and were treated with conversion therapy and surgery. Patients were categorized into responders (n=9) and non-responders (n=11) according to PTR. The parameters of PET/CT, including lesion size, SUVmean (mean standard uptake value), MTV (metabolic tumor volume), TLG (total lesion glycolysis), SUVpeak (peak standard uptake value), and TLR (tumor-to-normal liver standardized uptake value ratio), were calculated. The diagnostic efficacy was evaluated by receiver operating characteristic analysis (ROC). PTR was compared with pretreatment PET/CT parameters by using Spearman correlation analysis. The patients were followed up.

**Results:**

There was significant difference in TLR (5.59 ± 1.90 vs. 2.84 ± 1.70, respectively; *P*=0.003) between responders and non-responders, with the largest area under the curve (sensitivity=100%, specificity=72.7%, AUC=0.899, 95%CI: 0.759-1.000, optimal diagnostic threshold of 3.09). The relationship between ^18^F-FDG PET/CT parameters and PTR indicated TLR was moderately and positively correlated with pathological treatment response, with correlation coefficients (rs) of 0.69 (*P*<0.01). During the follow-up, no patients died, and tumor recurrence was found in one of the responders (11.1%). In all 11 non-responders, tumor recurrence was found in six patients (54.5%) and four patients (36.4%) died.

**Conclusions:**

TLR may be a powerful marker to predict PTR of HCC patients with BCLC stage C who were treated with conversion therapy.

## Introduction

Hepatocellular carcinoma (HCC) is the most common pathological type of liver cancer, accounting for 90% of primary liver cancers (1). Most HCC patients are diagnosed as local advanced or metastatic diseases, and patients may lose the opportunity for a radical cure with a poor prognosis (2). According to the criteria for the diagnosis and treatment of Barcelona Clinic Liver Cancer (BCLC) system (3) and the majority of international guidelines, patients with portal thrombosis or extrahepatic spread (BCLC stage C) should be treated with systemic therapies.

The current systemic therapy for patients with advanced HCC consists of tyrosine kinase inhibitors (TKI) and immune checkpoint inhibitors (ICI) (4). TKI, lenvatinib, as an emerging first-line systemic therapy for HCC, has been approved in China, USA, EU, Japan, and other countries for advanced HCC (5). Recent clinical studies have found that the combination of ICI and TKI was more effective. For example, the combination of lenvatinib with pembrolizumab or nivolumab showed objective response rates (ORRs) of 36% and 54.2%, respectively (6, 7). Another surprising discovery was that some patients who received ICI plus TKI as a conversion therapy retrieved the opportunity of surgery (8–14).

^18^F-fluorodeoxyglucose positron emission tomography (^18^F-FDG PET) is generally used for diagnosis, staging, and monitoring treatment of cancers, but shows poor sensitivity for the detection of HCC compared with other solid tumors (15). The higher ^18^F-FDG uptake appears to present in more undifferentiated tumors (16), and ^18^F-FDG uptake positive HCC is associated with a poor response to various treatments (17, 18). However, some studies found that ^18^F-FDG uptake is correlated with PD-1/PD-L1 status (19), and high ^18^F-FDG uptake may be a useful predictor of a rapid lenvatinib treatment response (20). Based on the findings above, we hypothesized that metabolic parameters of ^18^F-FDG PET may predict the patients who benefit from conversion therapy and may facilitate the choice of optimal therapy.

The main aim of this study was to evaluate the ability of metabolic parameters of pretreatment ^18^F-FDG PET to predict the pathological treatment response of HCC patients with BCLC stage C who were treated with PD-1 inhibitors plus lenvatinib.

## Materials and Methods

### Patients

Between July 2019 and April 2021, a total of 62 HCC patients with BCLC stage C who underwent pretreatment ^18^F-FDG PET/CT in Department of Nuclear Medicine, Chinese PLA General Hospital were retrospectively recruited.

The inclusion criteria were as follows:

1. All patients were aged >18 years without a history of other malignancies.
2. The diagnosis of HCC was confirmed by fine-needle biopsy pathology or in accordance with the clinical diagnosis criteria of the American Association for the Study of Liver Diseases (AASLD) (21).
3. All patients were diagnosed as HCC of BCLC stage C.
4. ^18^F-FDG PET/CT was performed within 2 weeks prior to treatment.
5. All patients were treated with PD-1 inhibitors plus lenvatinib after clinical evaluation in our hospital (Faculty of Hepato-Pancreato-Biliary Surgery, Chinese PLA General Hospital).
6. No other anti-tumor treatment was given during the treatment of PD-1 inhibitors plus lenvatinib, and the drugs were not stopped or changed during the treatment.
7. All patients received surgical treatment, and postoperative pathological examination was performed.

**Figure 1:**
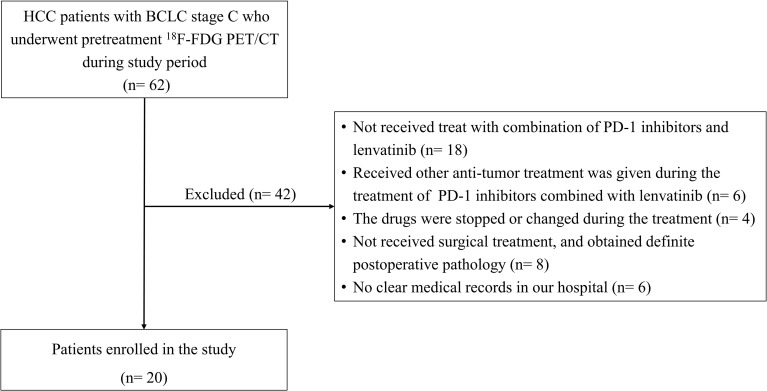
Patient in- and exclusion flow diagram. HCC, Hepatocellular carcinoma; BCLC, Barcelona Clinic Liver Cancer; ^18^F-FDG PET/CT, ^18^F-fluorodeoxyglucose positron emission tomography/computed tomography.

Finally, a total of 20 patients were included in this study (**Figure 1**). This single-center retrospective study was approved by the institutional ethics committee of the General Hospital of the People’s Liberation Army. All patients signed the informed consent before ^18^F-FDG PET/CT. The study was performed in accordance with the Declaration of Helsinki.

### PET/CT Scanning

All patients were scanned with ^18^F-FDG PET/CT (Biograph 64, GE Healthcare). Patients were fasted for 6 hours with plasma glucose levels < 11.1 mmol/L, and rested for at least 20 min in a quiet waiting room before intravenous administration of ^18^F-FDG (^18^F-FDG; Manufactured by our department, radiochemical purity of >95%). The patients were injected with ^18^F-FDG at a dose of 3.70-4.44 MBq/kg (0.10-0.12mCi/kg). PET/CT scan was performed after 60 min, beginning from the skull base to the upper femur in free-breathing mode. The low dose CT (LDCT) parameters: voltage=120kV, current=100mAs, rotation=0.8, layer thickness=5mm, pitch=1. The parameters of PET included three-dimensional mode, 2 min/bed (30% overlap), 4-5 beds/person, three iterations, 21 subsets, Gaussian filter half-height width=4.0 mm. The images were reconstructed with CT attenuation correction (AC) by using the ordered subset expectation maximization algorithm (OSEM).

### Image Analysis

Multiparametric Analysis prototype (GE Healthcare, Germany), a dedicated prototype post-processing tool, was used for imaging analysis. Quantitative analyses were performed by two experienced physicians of nuclear medicine blinded to the clinical information of the patients. If the views were inconsistent between the two physicians, the process would be repeated two weeks later until a consensus was reached. Areas with abnormal uptake of ^18^F-FDG on PET and/or abnormal density on CT were defined as lesions. A two-dimensional region of interest (ROI) was delineated manually according to the boundary of the tumor lesion on each layer of transaxial CT images to form a three-dimensional volume of interest (VOI). Sometimes contrast-enhanced magnetic resonance imaging (MRI)/CT were used to help determine the VOI. We applied the VOI to the corresponding PET images, which were registered to CT images. To measure normal liver activity, three non-overlapping spherical 1-cm^3^-sized VOIs were drawn in the normal liver on the axial PET images, avoiding the HCC areas on dynamic CT. MTV and TLG were obtained based on a threshold SUV of≥ 2.5.

The parameters of PET/CT, including lesion size (diameters, mm), SUVmean (mean standard uptake value), MTV (metabolic tumor volume), TLG (total lesion glycolysis, SUVmean×MTV), SUVpeak (peak standard uptake value), and TLR (tumor-to-normal liver standardized uptake value ratio, SUVmax of the tumor/SUVmean of the normal liver parenchyma), were calculated.

### Systemic Therapy

Conversion therapy mainly includes PD-1 inhibitors and lenvatinib. Lenvatinib was taken orally (8 or 12 mg/day, depending on patients’ weight<60kg or ≥60kg). The patients were treated with intravenous infusion of anti-PD-1 antibodies (dose, 200-240mg), and the great majority of the data collected were associated with four treatment regimens (pembrolizumab 200 mg/q3w, sintilimab 200 mg/q3w, toripalimab 240 mg/q3w, and tislelizumab 200mg/q3w), as shown in **Table S1**.

### Follow-Up During Systemic Therapy and Radiological Assessment

All patients were treated regularly and should be monitored to assess their response to systemic therapy. The complete blood count, thyroid, cardiac, liver, renal, adrenal functions, and tumor markers were examined prior to each cycle of PD-1 treatment. Before and after the conversion treatment protocol, imaging examinations were performed. For patients with multiple lesions, we defined the largest lesion as the main lesion by pretreatment contrast-enhanced MRI/CT and ^18^F-FDG PET/CT. Tumor response [according to RECIST v1.1 (22) and mRECIST criteria (23)] and the resectability of liver cancer were evaluated by contrast-enhanced MRI/CT, chest CT, and other liver function assessments. Adverse events were assessed using the National Cancer Institute’s Common Terminology Criteria for Adverse Events v4.0.

### Criteria for Successful Conversion Therapy

The criteria for successful conversion were below:

1. Child-Pugh grade A.
2. ECOG PS score ≤ 1.
3. Metastatic lymph nodes shrink or disappear, and the remaining lymph nodes can be removed.
4. No new extrahepatic metastases.
5. Intact vascular structure (including inflow and outflow) of the reserved liver.
6. The expected ratio of future liver remnant volume to standard liver volume (FLR/SLV) after resection of tumor-bearing liver is ≥40% in the compromised liver and 35% in the normal liver.

All patients who met the criteria of successful conversion were informed of the benefits and risks of surgery.

### Histopathologic Assessment of Tumor Regression

Surgical specimens were analyzed by two experienced pathologists who were blinded to the treatment and outcomes. The pathological treatment response was classified based on the tumor cellularity, and only the primary tumors were analyzed. Major pathologic response (MPR, <10% residual viable tumor cellularity) after conversion immunotherapy was used as an endpoint in the great majority of clinical trials (24). Hence, we divided all patients into two groups: responders (<10% cellularity), and non-responders (≥10% cellularity).

### Postoperative Therapy and Follow-Up

Patients continued to receive therapy according to the pathological results and personal conditions 4-6 weeks after surgery, and clinical evaluation. Serum tumor biomarkers were examined every cycle, and imaging examinations (contrast-enhanced MRI/CT or abdominal ultrasound) were performed every 3 months to monitor tumor recurrence. HCC recurrence was defined as the presence of radiological evidence of new intra- and/or extrahepatic tumors (25). According to the guidelines (26), post-recurrence treatments were administered.

### Statistical Analysis

Qualitative data were expressed as number of cases and percentage [n (%)]. Quantitative data were expressed as medians (interquartile ranges) or mean ± SD. The student *t* test or Mann-Whitney test was used to compare pretreatment ^18^F-FDG PET/CT parameters between responders and non-responders. The optimal cut-off values for continuous variables were estimated using receiver operating characteristic (ROC) curve analysis with the area under the curve (AUC), and we calculated sensitivity, specificity, positive predictive value (PPV), and negative predictive value (NPV), respectively. Pathological treatment response was compared with pretreatment ^18^F-FDG PET/CT parameters by using Spearman correlation analysis. The statistical analysis was performed by using commercially available software (IBM SPSS Statistics 24, IBM, Armonk, NY; and R software program, version 4.0.2, Bell Laboratories, USA). All statistical tests were two-sided, and the significance level was set at *P*=0.05.

## Results

### Patients’ Characteristics

Patient characteristics before treatment are shown in **Table 1**. Eventually, 20 patients underwent surgical excision after successful conversion therapy, who were enrolled in our study (18 male; median age, 54.0 years; IQR, 42.3–60.3 years). Eleven patients had a history of liver diseases (including 11 HBV, three HCV, and one alcoholic liver disease). The ECOG PS score of all cases was 0, and their liver function was Child-Pugh grade A before treatment.

**Table 1:** Baseline Clinical and Pathologic Characteristics.

Characteristic	Datum
**General status**	
** Age**	54.0 (42.3-60.3)
** Male : Female**	18:2
** Alcohol abuse**	11 (55%)
** History of liver diseases**	
** None**	5 (20%)
** Hepatitis B**	11 (50%)
** Hepatitis C**	3 (15%)
** ALD**	1 (5%)
** Body mass index**	
** Underweight (< 18.5kg/m^2^)**	1 (5%)
** Normal weight (18.5–25kg/m^2^)**	10 (50%)
** Overweight (25–30 kg/m^2^)**	8 (40%)
** Obese (>30kg/m^2^)**	1 (5%)
** ECOG PS score**	
** 0**	20 (100%)
** ≥1**	0 (0%)
**Clinical findings**	
** Liver function status**	
** Child-Pugh A**	20 (100%)
** Child-Pugh B**	0 (0%)
** AFP**	
** < 400**	9 (45%)
** ≥ 400**	11 (55%)
**Imaging findings**	
** Cirrhosis**	15 (75%)
** Tumor number**	
** Single**	13 (65%)
** Multiple**	7 (35%)
** Tumor diameter (mm)**	83.5 (66.3-112.3)
** Hepatic vein tumor thrombus**	17 (85%)
** Lymphatic metastasis**	10 (50%)
**Surgery findings**	
** Type of hepatectomy**	
** Anatomic resection**	19 (95%)
** Non-anatomic resection**	1 (5%)
** R0 resection**	20 (100%)
**Pathologic findings**	
** Major pathologic response**	
Responders	9 (45%)
** Non-responders**	11 (55%)

Data are medians with interquartile ranges or numbers of participants with percentages. ALD, Alcoholic liver disease; ECOG PS, Eastern Cooperative Oncology Group performance status; AFP, Alpha fetoprotein.

Sixteen of the included patients were treated with 3-6 cycles of PD-1 inhibitors plus lenvatinib, two patients received seven cycles, one patient received nine cycles, and one patient received 20 cycles. Nineteen patients received anatomic resection, and their surgical margins were all negative for malignancy (R0).

### The Value of Pretreatment ^18^F-FDG PET/CT Parameters in Prediction of Pathological Treatment Response

Among 20 patients, nine patients (45.0%) were considered responders. Pretreatment ^18^F-FDG PET/CT parameters were compared between responders and non-responders, and it was found that pretreatment TLR (5.59 ± 1.90 vs. 2.84 ± 1.70, respectively; *P*=0.003), SUVmax (13.56 ± 4.51 vs. 7.77 ± 4.78, respectively; *P*=0.013), SUVpeak (11.80 ± 3.77 vs. 6.94 ± 4.30, respectively; *P*=0.016), TLG [3942.3 (1293.4-7013.5) vs. 1148.4 (273.5-2357.6), respectively; *P*=0.025] and MTV [759.3 (303.4-1039.9) vs. 265.6 (77.3-533.4), respectively; *P*=0.030] were higher in the responder group than in the non-responder group. However, there was no significant difference in SUVmean between responder and non-responder groups (**Table 2**).

**Table 2:** The value of pretreatment ^18^F-FDG PET/CT parameters between responders and non-responders.

Parameter	Responders (n=9)	Non-responders (n=11)	*P*
**Lesion size (mm)**	107.22 ± 39.14	75.09 ± 30.70	0.054^*^
**SUVmax**	13.56 ± 4.51	7.77 ± 4.78	0.013^*^
**SUVmean**	5.33 ± 1.49	3.84 ± 1.47	0.146^*^
**TLG**	3942.3 (1293.4-7013.5)	1148.4 (273.5-2357.6)	0.025^#^
**MTV**	759.3 (303.4-1039.9)	265.6 (77.3-533.4)	0.030^#^
**SUVpeak**	11.80 ± 3.77	6.94 ± 4.30	0.016^*^
**TLR**	5.59 ± 1.90	2.84 ± 1.70	0.003^*^

Data are medians with interquartile ranges in parentheses or mean ± SD.

*Student t test; ^#^Mann-Whitney test.

SUVmean, Mean standard uptake value); MTV, Metabolic tumor volume; TLG, Total lesion glycolysis; SUVpeak, Peak standard uptake value; TLR, Tumor-to-normal liver standardized uptake value ratio. Bold value: the most statistically significant parameter among all pretreatment 18F-FDG PET/CT parameters.

**Figure 2:**
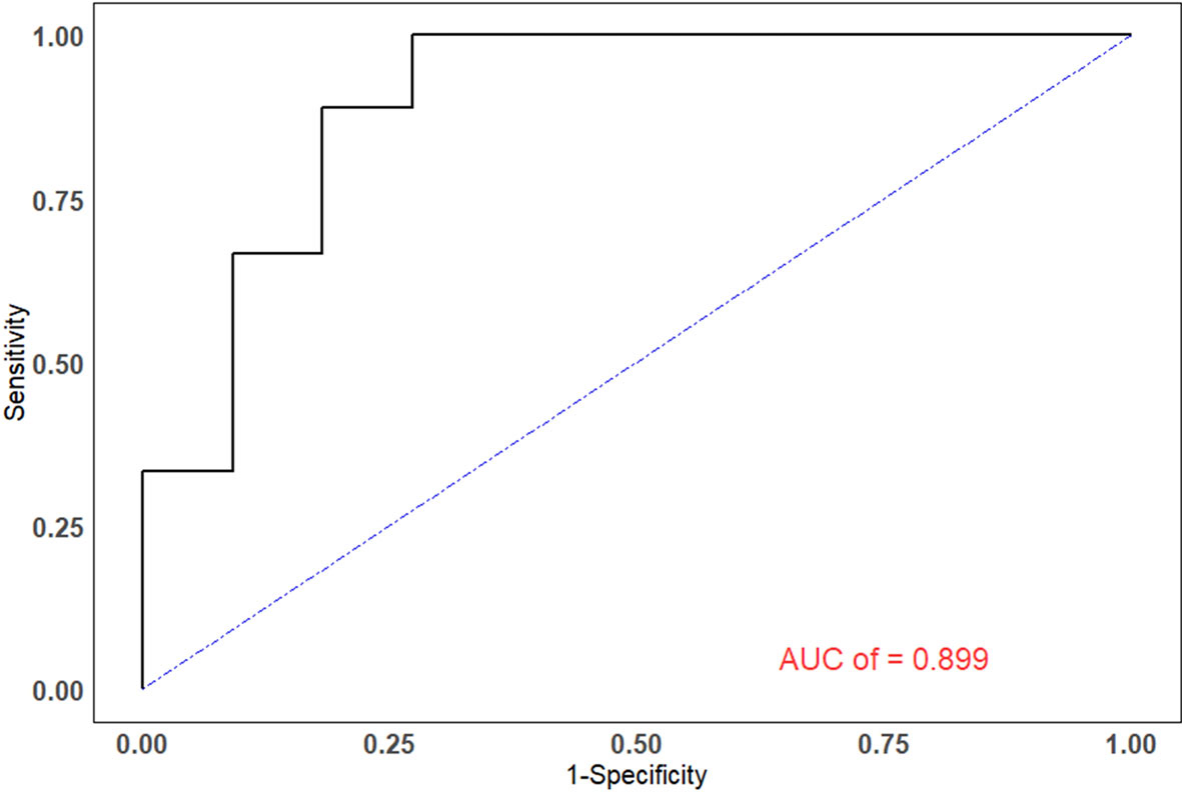
The ROC curves of TLR. The areas under the ROC curves for the ability to predict pathological treatment response for TLR was 0.899.

The efficiencies of lesion size and ^18^F-FDG PET/CT parameters for predicting pathological treatment response were evaluated. TLR showed the largest area under the curve (AUC=0.899, 95% confidence interval [CI]: 0.759-1.000), with a Youden index of 0.727, and optimal diagnostic threshold of 3.09. The positive predictive value (PPV) and negative predictive value (NPV) were 0.750 (0.428-0.933) and 1.000 (0.598-1.000), respectively. Details are shown in **Table 3** and **Figure 2**.

**Table 3:** Differential diagnostic efficiency of TLR between responders and non-responders.

Parameter	Cut-off	AUC	Sensitivity	Specificity	PPV	NPV
**TLR**	3.09	0.899 (0.759-1.000)	1.000 (0.629-1.000)	0.727 (0.393-0.927)	0.750 (0.428-0.933)	1.000 (0.598-1.000)

AUC, Area under the curve; PPV, Positive predictive value; NPV, Negative predictive value; TLR, Tumor-to-normal liver standardized uptake value ratio.

### Correlation Between ^18^F-FDG PET/CT Parameters and Pathological Treatment Response

**Figure 3:**
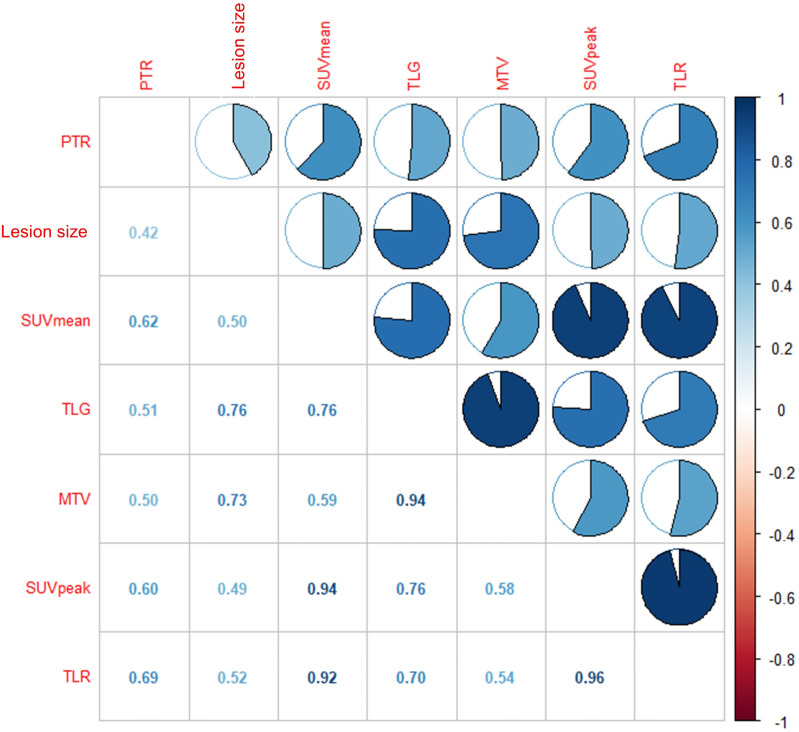
The results showed that moderate correlation between TLR and pathological treatment response (r = 0.69, *P* < 0.01), SUVmean and pathological treatment response (r = 0.62, *P* < 0.05), SUVpeak and pathological treatment response (r = 0.60, *P* < 0.05), respectively.

We performed Spearman correlation to explore the relationship between ^18^F-FDG PET/CT parameters and pathological treatment response. The results showed that TLR, SUVmean, and SUVpeak had moderate positive correlation with pathological treatment response, and the correlation coefficients (rs) were 0.69, 0.62, and 0.60, respectively (*P*<0.05). Lesion size, TLG, and MTV showed no significant correlation (r =0.42, r =0.51 and r =0.50, respectively; *P*>0.05). The results are displayed in **Table 4** and **Figure 3**.

**Table 4:** The correlation between pretreatment ^18^F-FDG PET/CT parameters and pathological treatment response.

Parameter	Spearman’s rho coefficient
**Lesion size**	r=0.42
**SUVmean**	r=0.62*
**TLG**	r=0.51
**MTV**	r=0.50
**SUVpeak**	r=0.60*
**TLR**	r=0.69**

*P < 0.05, **P < 0.01. SUVmean, Mean standard uptake value); MTV, Metabolic tumor volume; TLG, Total lesion glycolysis; SUVpeak, Peak standard uptake value; TLR, Tumor-to-normal liver standardized uptake value ratio.

### Follow-Up

The follow-up ended on January 1, 2022. No patient was lost to follow-up and the median follow-up time was 15.8 months (range, 5.0-32.9 months). During the follow-up, all nine responders remained alive, and tumor recurrence was found in two patients (2.2%; at 6.7 months and 9.9 months, respectively). However, in the non-responder group, tumor recurrence was found in six patients [54.5%; a median of 5.9 months (range, 4.6-14.4 months)] and four patients [36.4%; a median of 10.3 months (range: 8.2-12.9 months)] died. Follow-up information of the patients is shown in **Table S2**.

## Discussion

This is the first study that utilized the metabolic parameters of pretreatment ^18^F-FDG PET to predict the pathological treatment response of HCC patients with BCLC stage C who were treated with PD-1 inhibitors and lenvatinib. Our findings showed that the metabolic parameters of pretreatment ^18^F-FDG PET could distinguish responders from non-responders, and a high TLR (TLR≥3.09) was likely to predict pathological treatment response. This result may suggest the pretreatment ^18^F-FDG PET can help HCC patients of BCLC stage C stratified before treatment of PD-1 inhibitors and lenvatinib.

Surgery has always been the most effective treatment for liver cancer. However, patients with advanced HCC (BCLC stage C) often have lost the opportunity for surgery, and systemic treatment is recommended by the guidelines (1, 27). TKI, such as sorafenib and lenvatinib, has been proven effective in the first-line systemic therapy of advanced HCC patients (5, 28). In recent years, ICI (anti-PD-1, anti-PD-L1, and anti-CTLA-4 antibodies) has exhibited potential therapeutic effects for advanced HCC (29). Although the previously discussed approaches provide novel opportunities for the treatment of advanced HCC, satisfactory clinical results cannot be obtained using single therapy. Some studies have shown that the combination of PD-1/PD-L1 inhibitors and anti-angiogenesis targeted drugs showed a better curative effect (6, 7, 30–33). For example, lenvatinib combined with pembrolizumab or nivolumab showed promising antitumor activity with a tolerable safety profile in patients with advanced HCC (6, 7). Of note, Ho et al. reported a case of successful conversion of locally advanced hepatocellular carcinoma to resectable tumor after treatment with TKI plus immunotherapy (11). Since then, Ho et al. pursued research, and it was found that cabozantinib and nivolumab converted locally advanced hepatocellular carcinoma into resectable disease (12). Of 20 patients enrolled, 20 (100%) underwent successful margin-negative resection (R0) and nine out of 20 (45%) had MPR. Zhu et al. found the combination of TKI/anti-PD-1 antibodies was a feasible conversion therapy for patients with unresectable HCC to become resectable (13). A prospective clinical registration study (ChiCTR1900023914) also found that patients who were treated with PD-1 inhibitors plus TKI were successfully converted and subsequently underwent surgery (8–10). Therefore, we conducted a preliminary study on the relationship between parameters of pretreatment ^18^F-FDG PET and pathological treatment response.

Pretreatment FDG PET can provide useful information on the expression of immune checkpoints and the metabolic state of the tumor microenvironment (34), and ^18^F-FDG uptake is correlated with the expression of PD-1/PD-L1 in lung cancer and bladder cancer (35–37). However, HCC is a very heterogeneous tumor from a genetic standpoint with different oncogenic pathways (38). Immunotherapy based on checkpoint inhibitors in HCC is not associated with increased or decreased efficacy based on PD-1 or PD-L1 assessment (39), and only serum alpha-fetoprotein (AFP) levels might play a clinical role in patients treated by TKI (40). In our study, the metabolic parameters of pretreatment ^18^F-FDG PET could distinguish responders from non-responders. One explanation is that ^18^F-FDG uptake and tumor infiltrative lymphocytes (TILs), especially T cells, might be the causes of this phenomenon (41, 42). Itoh et al. found that SUVmax values of HCC were associated with intratumoral CD8^+^ T cell counts (*P*=0.0044) (43). It was further found that immune cells, including T cells and B cells, were significantly enriched in tumor cells of locally advanced hepatocellular carcinoma patients responding to the novel combination of cabozantinib and nivolumab (11, 12). Moreover, Kawamura et al. reported that high TLR≥2 in HCC may be a useful predictor of an extremely rapid response to lenvatinib (20). The evidence above may suggest that high ^18^F-FDG uptake of tumors is related to the effectiveness of PD-1 and TKI treatment. Our results suggest that a high TLR (TLR≥3.09) can be used as a powerful indicator to predict the pathological treatment response of HCC patients of BCLC stage C who were treated with PD-1 inhibitors and lenvatinib as a conversion therapy (**Figures 4A**, **B**). It is noteworthy that, although high TLR may suggest a response to the combination of PD-1 inhibitors and lenvatinib, ^18^F-FDG PET positive HCC is highly associated with poor histological differentiation and poor prognosis (20, 44). But other exploratory analyses showed that higher expression of PD-L1 in tumor tissues, higher expression of vascular endothelial growth factor (VEGF) receptor 2, and higher T-regulatory cells immune phenotype may be associated with longer survival of patients treated by atezolizumab plus bevacizumab (45). It is suggested that ^18^F-FDG PET may direct systemic treatment of patients with advanced HCC (1). However, biomarkers related to the treatment of advanced HCC are still very limited, and this conjecture remains to be proven by more larger studies.

**Figure 4A:**
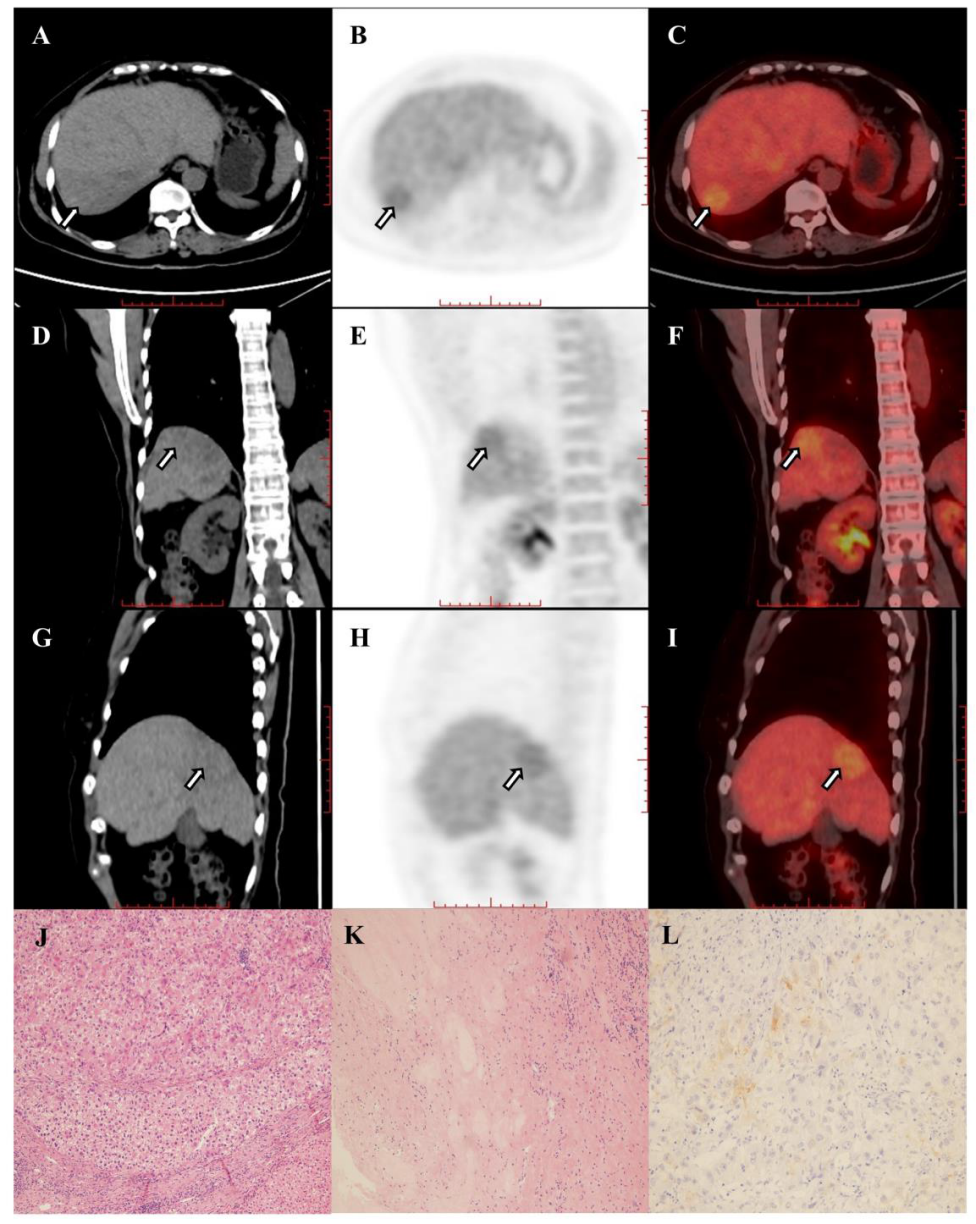
**(A–I)** Images in 67-year-old woman with hepatocellular carcinoma in the right hepatic lobe (arrow), and patient was accompanied by portal vein tumor thrombus and hilar lymph node metastasis. The hepatic lesion showed low uptake (TLR=1.31). The baseline AFP was 2.19 and the Child-Pugh score was 6. The patient underwent right hemihepatectomy after 4 cycles of combination of pembrolizumab plus lenvatinib therapy. Histopathologic evaluation of response revealed bad response to therapy (residual viable tumor cellularity>90%, **J–L**).

**Figure 4B:**
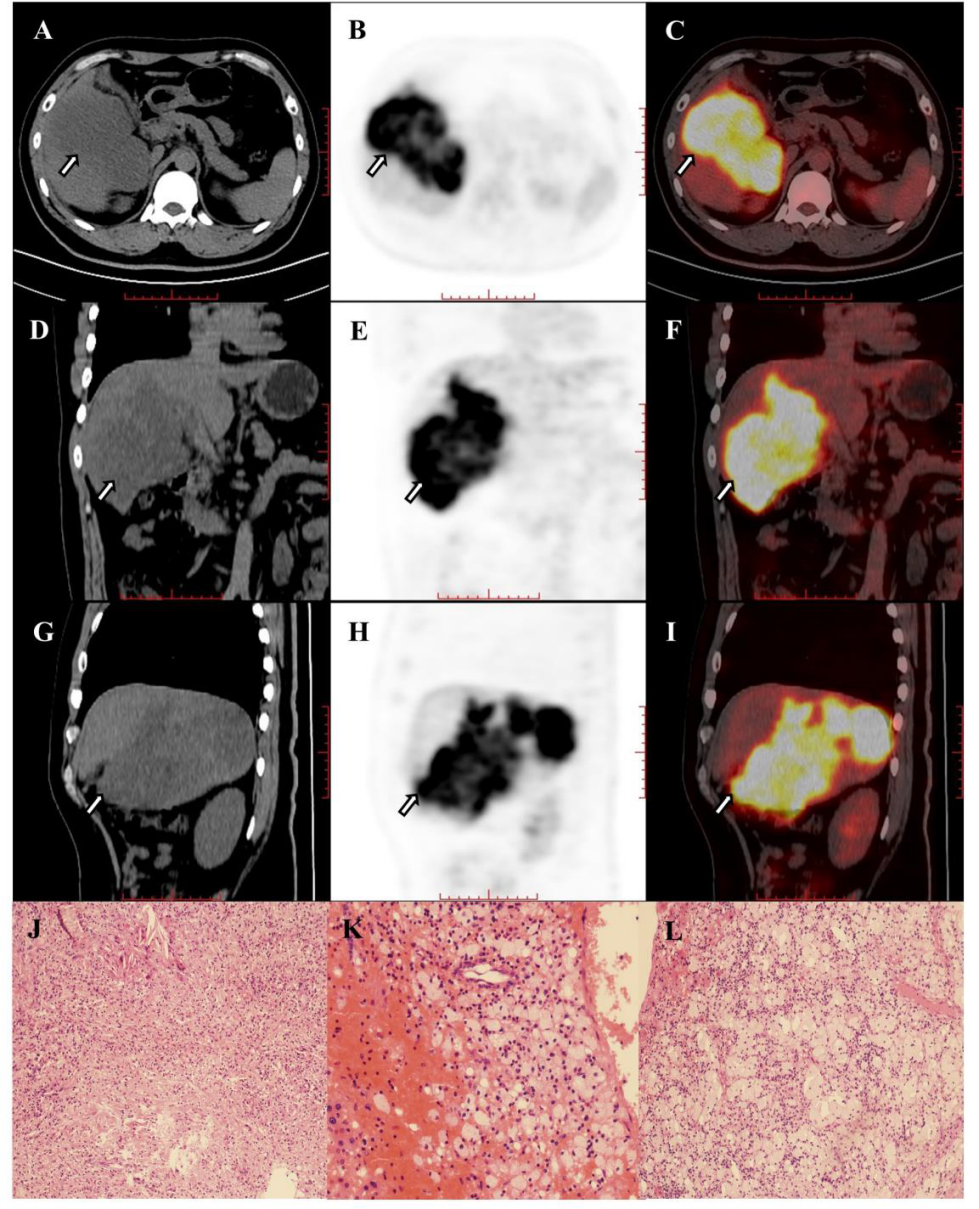
**(A–I)** Images in 42-year-old man with hepatocellular carcinoma in the right hepatic lobe (arrow) and hilar lymph node metastasis. The hepatic lesion showed high uptake (TLR=7.66). The baseline AFP was >60500 and the Child-Pugh score was 6. The patient underwent right hemihepatectomy after 4 cycles of combination of pembrolizumab plus lenvatinib therapy. Histopathologic evaluation of response revealed good response to therapy (complete disappearance of the tumor cells, **J–L**).

There are some limitations in this preliminary study. First, the number of patients was small in this retrospective and single-center cohort study. Second, only pathological treatment response of the primary tumor without venous thrombosis and peripheral lymph nodes was evaluated. However, our finding remains to be verified in the tumor with lymph node metastasis or distal metastasis. Third, we investigated the correlation between PET parameters and histopathological indexes, but due to insufficient number of patients, the correlation between PET parameters and progression-free survival or overall survival was not evaluated. Using the surgical specimen of the treated tumor, it is feasible to assess treatment efficacy and potentially predict disease-free survival and overall survival (24), and our limited follow-up results also indicate that responders had a better prognosis. Our study may provide evidence for the treatment of advanced HCC patients with ICI plus TKI. However, our findings remain to be further verified by a prospective study with a larger cohort and comprehensive evaluation of pathological treatment response and prognosis.

## Conclusion

The metabolic parameters of pretreatment ^18^F-FDG PET, especially a high TLR, may be a powerful marker to predict the pathological treatment response of HCC patients of BCLC stage C who were treated with PD-1 inhibitors and lenvatinib as a conversion therapy. The parameters of pretreatment ^18^F-FDG PET might be useful for making treatment strategies for HCC patients.

## Data Availability Statement

The raw data supporting the conclusions of this article will be made available by the authors, without undue reservation.

## Author Contributions

Conception and design: GW, WZ, SL, JT, GM, and BX; Data collation: GW, WZ, JC, XL, and ZW; Statistical Analysis: GW, YW, and ZG; Article writing: GW, WZ, JC, XX, and ZG; Article revision: YW, SY, SL, JT, GM, and BX. All authors contributed to the article and approved the submitted version.

## Conflict of Interest

YW was employed by the company GE Healthcare.

The remaining authors declare that the research was conducted in the absence of any commercial or financial relationships that could be construed as a potential conflict of interest.

## Publisher’s Note

All claims expressed in this article are solely those of the authors and do not necessarily represent those of their affiliated organizations, or those of the publisher, the editors and the reviewers. Any product that may be evaluated in this article, or claim that may be made by its manufacturer, is not guaranteed or endorsed by the publisher.
