# Supplementary material for: Pretreatment Metabolic Parameters Measured by 18F-FDG PET to Predict the Pathological Treatment Response of HCC Patients Treated With PD-1 Inhibitors and Lenvatinib as a Conversion Therapy in BCLC Stage C
Source: Front Oncol. 2022 Jun 3;12:884372. doi: 10.3389/fonc.2022.884372 (PMC9204225; doi:10.3389/fonc.2022.884372)
Supplement: Supplementary file 1 [file Table_1.docx]

**Table S1. Conversion** **therapeutic regimen for details**

| Patient No. | Therapeutic regimen | Treatment cycles | | Days from systemic therapy to surgery | Therapy  adverse events | Responder/  Non-responder |
| --- | --- | --- | --- | --- | --- | --- |
| 1 | Pembrolizumab + Lenvatinib | 4 | 94 | | - | Non-responder |
| 2 | Sintilimab + Lenvatinib | 7 | 138 | | Elevated blood pressure | Non-responder |
| 3 | Tislelizumab + Lenvatinib | 5 | 109 | | - | Responder |
| 4 | Sintilimab + Lenvatinib | 5 | 103 | | - | Non-responder |
| 5 | Pembrolizumab + Lenvatinib | 4 | 105 | | Elevated blood pressure | Responder |
| 6 | Sintilimab + Lenvatinib | 4 | 77 | | Skin rash | Non-responder |
| 7 | Toripalimab + Lenvatinib | 5 | 120 | | - | Non-responder |
| 8 | Sintilimab + Lenvatinib | 3 | 84 | | Skin rash | Non-responder |
| 9 | Sintilimab + Lenvatinib | 6 | 139 | | Diarrhea | Responder |
| 10 | Sintilimab + Lenvatinib | 7 | 150 | | - | Responder |
| 11 | Sintilimab + Lenvatinib | 9 | 202 | | - | Non-responder |
| 12 | Sintilimab + Lenvatinib | 4 | 73 | | - | Non-responder |
| 13 | Sintilimab + Lenvatinib | 5 | 103 | | - | Responder |
| 14 | Sintilimab + Lenvatinib | 3 | 133 | | - | Non-responder |
| 15 | Pembrolizumab + Lenvatinib | 4 | 73 | | - | Responder |
| 16 | Sintilimab + Lenvatinib | 4 | 92 | | - | Non-responder |
| 17 | Sintilimab + Lenvatinib | 3 | 69 | | Elevated blood pressure/ Liver dysfunction | Responder |
| 18 | Sintilimab + Lenvatinib | 20 | 525 | | - | Responder |
| 19 | Sintilimab + Lenvatinib | 4 | 100 | | - | Responder |
| 20 | Sintilimab + Lenvatinib | 6 | 167 | | - | Non-responder |

**Table S2.**  **The patient’s follow-up information**

| Patient No. | Recurrence | Days from systemic therapy to recurrence | Death | Cause Of Death | Days from systemic therapy to death | Responder/  Non-responder |
| --- | --- | --- | --- | --- | --- | --- |
| 1 | Recurrence | 196 | Death | Lung cancer | 366 | Non-responder |
| 2 | Recurrence | 169 | Death | Gastrointestinal bleeding | 249 | Non-responder |
| 3 | - | - | - | - | - | Responder |
| 4 | Recurrence | 139 | Death | Recurrence | 387 | Non-responder |
| 5 | Recurrence | 200 | - | - | - | Responder |
| 6 | Recurrence | 187 | - | - | - | Non-responder |
| 7 | Recurrence | 168 | - | - | - | Non-responder |
| 8 | Recurrence | 432 | - | - | - | Non-responder |
| 9 | - | - | - | - | - | Responder |
| 10 | - | - | - | - | - | Responder |
| 11 | - | - | - | - | - | Non-responder |
| 12 | - | - | - | - | - | Non-responder |
| 13 | - | - | - | - | - | Responder |
| 14 | - | - | Death | Respiratory failure | 245 | Non-responder |
| 15 | - | - | - | - | - | Responder |
| 16 | - | - | - | - | - | Non-responder |
| 17 | - | - | - | - | - | Responder |
| 18 | - | - | - | - | - | Responder |
| 19 | - | - | - | - | - | Responder |
| 20 | - | - | - | - | - | Non-responder |
